# Altered Plasma Fatty Acids Associate with Gut Microbial Composition in Common Variable Immunodeficiency

**DOI:** 10.1007/s10875-021-01146-9

**Published:** 2021-10-20

**Authors:** Tonje Skarpengland, Magnhild E. Macpherson, Johannes R. Hov, Xiang Y. Kong, Pavol Bohov, Bente Halvorsen, Børre Fevang, Rolf K. Berge, Pål Aukrust, Silje F. Jørgensen

**Affiliations:** 1 Research Institute of Internal Medicine, Oslo University Hospital Rikshospitalet, Oslo, Norway; 2 Section of Clinical Immunology and Infectious Diseases, Oslo University Hospital Rikshospitalet, Oslo, Norway; 3 Institute of Clinical Medicine, University of Oslo, Oslo, Norway; 4 Norwegian PSC Research Center, Division of Surgery, Inflammatory Diseases and Transplantation, Department of Transplantation Medicine, Oslo University Hospital Rikshospitalet, Oslo, Norway; 5 Section of Gastroenterology, Division of Surgery, Inflammatory Diseases and Transplantation, Department of Transplantation Medicine, Oslo University Hospital, Oslo, Norway; 6 Department of Clinical Science, University of Bergen, Bergen, Norway; 7 Department of Heart Disease, Haukeland University Hospital, Bergen, Norway

**Keywords:** ALA, ARA, alpha diversity, alpha-linolenic acid, arachidonic acid, *Bifidobacterium*, *Blautia*, CVID, common variable immunodeficiency, DHA, desulfovibrionaceae, docosahexaenoic acid, dysbiosis, EPA, eicosapentaenoic acid, fatty acids, hypogamma, IgG, immunoglobulin G, gut microbiota, LA, linoleic acid, MUFA, monounsaturated fatty acid, n-3 PUFA, n-6 PUFA, omega 3, omega 6, PID, polyunsaturated fatty acid, PUFA, rifaximin, ruminococcaceae

## Abstract

**Purpose:**

Fatty acid (FA) abnormalities are found in various inflammatory disorders and have been related to disturbed gut microbiota. Patients with common variable immunodeficiency (CVID) have inflammatory complications associated with altered gut microbial composition. We hypothesized that there is an altered FA profile in CVID patients, related to gut microbial dysbiosis.

**Methods:**

Plasma FAs were measured in 39 CVID patients and 30 healthy controls. Gut microbial profile, a food frequency questionnaire, and the effect of the oral antibiotic rifaximin were investigated in CVID patients.

**Results:**

The *n-3* polyunsaturated fatty acids (PUFAs), eicosapentaenoic acid (EPA) (1.4 [1.0–1.8] vs. 1.9 [1.2–2.5], median (IQR), *P* < 0.05), and docosahexaenoic acid (DHA) (3.2 [2.4–3.9] vs. 3.5 [2.9–4.3], *P* < 0.05), all values expressed as weight percent of total plasma FAs, were reduced in CVID compared to controls. Also, *n-6* PUFAs (34.3 ± 3.4 vs. 37.1 ± 2.8, mean ± SD, *P* < 0.001) and linoleic acid (LA) (24.5 ± 3.3 vs. 28.1 ± 2.7, *P* < 0.0001) and the FA anti-inflammatory index (98.9 [82.1–119.4] vs. 117.0 [88.7–153.1], median (IQR), *P* < 0.05) were reduced in CVID. The microbial alpha diversity was positively associated with plasma *n-6* PUFAs (*r* = 0.41, *P* < 0.001) and LA (*r* = 0.51, *P* < 0.001), but not *n-3* PUFAs (*P* = 0.78). Moreover, a 2-week course of rifaximin significantly reduced the proportion of *n-6* PUFAs (*P* = 0.04, UNIANOVA). Serum immunoglobulin G (IgG) levels correlated with plasma *n-3* PUFAs (rho = 0.36, *P* = 0.03) and DHA (rho = 0.41, *P* = 0.009).

**Conclusion:**

We found a potentially unfavorable FA profile in CVID, related to low IgG levels. High plasma *n-6* PUFAs were related to increased gut microbial diversity and altered by rifaximin therapy.

**Supplementary Information:**

The online version contains supplementary material available at 10.1007/s10875-021-01146-9.

## Introduction

Common variable immunodeficiency (CVID) has a prevalence of 1:25 000 to 1:50 0000 in Caucasians and is the most common symptomatic primary immunodeficiency in adults [1]. Approximately 10% of CVID patients have a monogenic cause [2], whereas the remaining patients have a more complex pattern of inheritance involving multiple genetic and environmental factors [3]. CVID comprises a heterogeneous group of patients characterized by hypogammaglobulinemia, particularly reduced immunoglobulin G (IgG), which results in recurrent infections with capsulated bacteria in the respiratory tract. In addition, a large proportion of the patients (70–80%) have non-infectious autoimmune and/or inflammatory complications, such as splenomegaly, lymphadenopathy, enteropathy, granulomas and lymphoid interstitial pneumonitis [3, 4]. The etiology of these inflammatory and autoimmune complications is largely unknown, but we have previously shown an association with altered gut microbial composition and blood lipids [5, 6].

**Fig. 1:**
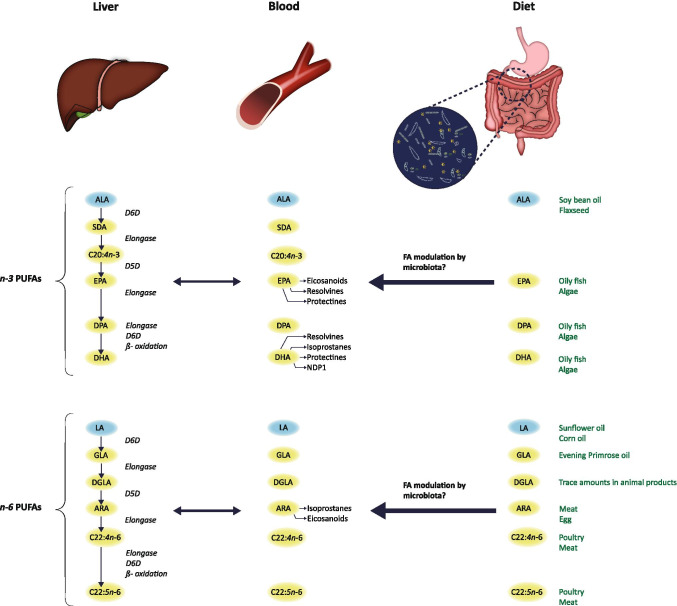
Metabolism and biosynthesis of *n-3* and *n-6* PUFAs in relation to diet and gut microbiota. This is a simplified illustration of factors influencing the most abundant plasma PUFAs, showing *n-3* and *n-6* PUFA biosynthesis and metabolism, as well as diet sources (green color) and the possible role of gut microbiota modulation of dietary fatty acids. ALA and LA (blue color) are essential fatty acids, obtained through diet only. The biosyntheses of non-essential PUFAs are mainly confined to the liver, but conversion rates are generally low. EPA, DHA, and DGLA are precursors of bioactive anti-inflammatory compounds (small arrows), and ARA gives rise to mostly inflammatory compounds (small arrows). ALA, alpha-linolenic acid; EPA, eicosapentaenoic acid; DPA, docosapentaenoic acid; DHA, docosahexaenoic acid; LA, linoleic acid; GLA, gamma-linolenic acid; DGLA, dihomo-gamma-linolenic acid; ARA, arachidonic acid; SDA, stearidonic acid; D5D, delta-5 desaturase; D6D, delta-6 desaturase. Illustration: Ine Eriksen, University of Oslo

Fatty acids (FAs), both free and as part of complex lipids, play a number of key roles in metabolism and cellular functions. They constitute a major component of cell membranes, act as precursors of extracellular signaling molecules, and influence membrane-derived intracellular signaling pathways. These factors are important for human health and disease risk, and numerous studies support a role for FAs in various autoimmune and inflammatory disorders [7, 8]. In general, the *n-3* polyunsaturated FAs (PUFAs) eicosapentaenoic acid (EPA) and docosahexaenoic acid (DHA) seem to have anti-inflammatory properties, whereas the *n-6* PUFA arachidonic acid (ARA) appears to mediate inflammatory reactions, at least partly by acting as precursors for various bioactive lipid mediators with opposing biological effects [9] (Fig. 1). Circulating FAs are also used as biomarkers of dietary FA intake in epidemiological studies, often in relation to inflammatory or cardiometabolic outcomes [10, 11]. However, accumulating evidence supports gut microbiota as a modifier of the dietary impact on the host metabolic state, including FA metabolism [12], linking gut microbiota to metabolic and inflammatory diseases.

Since a large proportion of patients with CVID have autoimmune and/or inflammatory complications, as well as a disturbed gut microbiota and intestinal pathology, we hypothesized that the plasma FA profile is altered in CVID patients and related to gut microbial dysbiosis rather than diet. In the present study, we therefore evaluated the plasma FA composition in CVID patients compared with healthy controls, and its relation to gut microbial composition, diet, and immunological parameters. We also analyzed the effect of a gut microbiota-targeted intervention on the plasma FA profile after a 2-week course of the non-absorbable antibiotic rifaximin.

## Methods

### Participants and Procedure

CVID was defined as decreased serum levels of IgG, in addition to IgA and/or IgM by a minimum of two standard deviations below the mean for age, and exclusion of other causes of hypogammaglobulinemia [13]. CVID subgroups were classified as “complications” (i.e., presence of one or more of the following complications: splenomegaly, lymphoid hyperplasia, granulomas, enteropathy, organ-specific autoimmunity, autoimmune cytopenia, lymphoid interstitial pneumonitis, nodular regenerative hyperplasia, or lymphoma) or as “infection only” (i.e., only recurrent bacterial infections in the respiratory tract and absence of the above mentioned complications), based on previously defined criteria [14]. Importantly, none of the patients in the “infection only” group had ongoing acute infection as this was an exclusion criterion. CVID enteropathy was defined as persistent diarrhea after exclusion of gastrointestinal infection [5].

This study is a secondary data analysis of a randomized controlled trial that analyzed the effect of a 2-week course oral rifaximin on systemic inflammation and gut microbiota composition [15]. The majority of the analyses was performed on plasma, diet, and gut microbiota data from the CVID baseline cohort (before randomization), collected between Oct 8, 2013, and Oct 20, 2014. The effects of rifaximin on FAs are presented at the end of the paper. Details regarding the randomization, masking, and procedures of the rifaximin trial have previously been described [15] but are also given in Supplemental methods.

In the present study, 39 CVID participants were compared to 30 healthy controls (one patient from the original rifaximin study was not included because there was not enough plasma available for FA analyses). The overall exclusion criteria were ongoing infection, antibiotics in the last 12 weeks, a history of allergic reaction to rifaximin, malignancy, impaired kidney function, pregnancy or lactation, use of probiotics in the last 6 months, immunosuppressive drugs, comorbidity that could influence with the patient’s safety or compromise the study results (e.g., cardiovascular disorders, alcoholism, psychiatric disease, HIV infection), and polypharmacy (patient with an extensive medication list, i.e., ten drugs or more). Exclusion of patients with polypharmacy was due to the anticipated effect on microbiota composition [16, 17].

The Regional Committee for Medical and Research Ethics approved the study protocol number 2013/1037. All study participants signed a written, informed consent. The work described herein has been carried out in accordance with the Declaration of Helsinki.

### Blood Sampling Protocol

Non-fasting peripheral venous blood was drawn into sterile blood collection tubes with EDTA as anticoagulant (plasma). The tubes were immediately immersed in melting ice, centrifuged within 15 min at 2000* g* for 20 min to obtain platelet-poor plasma. Plasma was stored at − 80 °C in aliquots and thawed only once. Method for flow cytometry analyses of lymphocyte subpopulations is previously described [15]. For flow cytometric evaluation of B cells, patients were categorized according to EUROclass classification [18]. IgG levels were measured in serum by immunturbidimetry (Roche, Basel, Switzerland) at the Department of Medical Biochemistry, Oslo University Hospital Rikshospitalet, Oslo, Norway.

### Plasma FA Composition

The total FA composition was analyzed in EDTA-plasma, as previously described [19]. FA concentrations are expressed as percentages of total FAs by weight (wt%). The *omega 3* index was defined as the sum of EPA and DHA, expressed as a percentage of the total FA content. The anti-inflammatory index was defined as the sum of EPA (C20:5*n-3*), DGLA (C20:3*n-6*), DHA (C22:6*n-3*), and DPA (C22:5*n-3*), divided on ARA (C20:4*n-6*), multiplied with 100 as previously described [20, 21].

### Stool Collection and Analysis

Participants collected stool samples at home within 24 h prior to their hospital visit, or alternatively at the hospital, with a standardized collection device [22]. The stool samples were then transferred by the participants to stool collection tubes with Stool DNA Stabilizer (Stratec Biomedical, Birkenfeld, Germany) [23]. Samples were stored at minimum − 20 °C according to the manufacturer’s recommendations until DNA extraction. Bacterial DNA was extracted using the PSP Spin Stool DNA Plus Kit (Stratec) before being subjected to amplification of the 16S ribosomal RNA gene with dual-indexed barcodes according to an established protocol [24], followed by sequencing on an Illumina MiSeq (San Diego, CA; Supplemental Methods).

### Food Frequency Questionnaire

CVID patients were asked to complete a self-administrated, validated Norwegian food frequency questionnaire designed to reflect dietary habits over the past year [25, 26]. The questionnaire offers multiple-choice alternatives and the opportunity to provide supplemental information regarding specific dietary restrictions or habits. It covers 180 food items and has serving size alternatives specified in household units, which is then converted to grams per day using software developed at the Institute for Nutrition Research, University of Oslo [25]. Thirty-seven CVID patients completed the questionnaire.

### Statistical Analysis

Univariate analyses were performed using parametric (*t*-test) or non-parametric methods (Mann–Whitney *U*) for continuous variables, and Fisher’s exact test for categorical variables, as appropriate. Correlation analyses were performed using parametric (Pearson) or non-parametric (Spearman) tests as appropriate. Comparisons between dichotomous variables were not made if one of the groups consisted of less than five individuals. Univariate repeated measures ANOVA (UNIANOVA) was used to assess the effect of treatment focusing on the interaction between time and treatment group for the different FAs, followed by paired samples *t*-test or Wilcoxon’s rank-sum test for paired data if significant. For the longitudinal data, we compared datasets from three different time points using the ANOVA test (parametric) or Friedman’s test (non-parametric). Calculations were performed in SPSS (version 24, IBM, NY).

## Results

### Reduced EPA and DHA in Patients with CVID

**Fig. 2:**
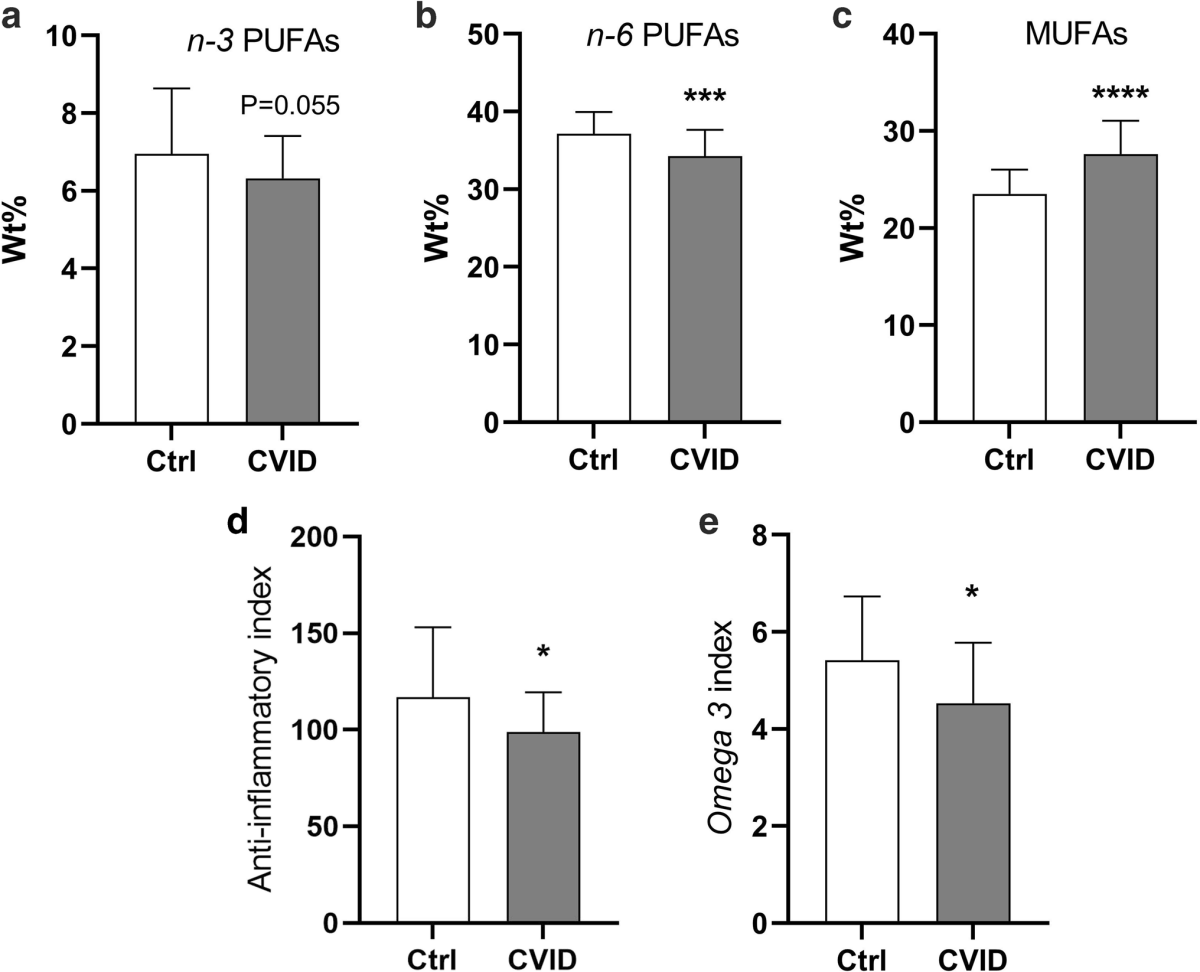
FA profile in CVID and healthy controls. Plasma FA proportions of **A** *n-3* PUFAs, **B** *n-6* PUFAs, **C** MUFAs, **D** anti-inflammatory index, and **E** *omega 3* index in controls (Ctrl, *n* = 30) and patients with CVID (common variable immunodeficiency, *n* = 39). The anti-inflammatory index was defined as the sum of EPA (C20:5*n-3*), DGLA (C20:3*n-6*), DHA (C22:6*n-3*), and DPA (C22:5*n-3*), divided on ARA (C20:4*n-6*), multiplied with 100. The *omega 3* index was defined as the sum of EPA and DHA, expressed as a percentage of the total FA content. Data are presented with mean (SD) or median (25–75 percentile) and were analyzed using the Mann–Whitney *U* test or Student’s *t*-test, as appropriate. PUFAs, polyunsaturated fatty acids; MUFAs, monounsaturated fatty acids; Wt%, weight %. *P < 0.05, **P < 0.01, ***P < 0.001, and ****P < 0.0001 versus controls

Baseline characteristics of patients (*n* = 39) and controls (*n* = 30) are presented in Table 1, showing no significant differences in age, sex, BMI, statin use, or smoking. We found reduced proportions of the two most abundant *n-3* PUFAs, EPA (*P* < 0.05), and DHA (*P* < 0.05), both with potential anti-inflammatory effects, in patients with CVID compared to controls (Table 2), with a non-significant trend towards lower total proportion of *n-3* PUFAs in CVID patients (*P* = 0.055, Fig. 2A).

**Table 1:** Baseline characteristics of the study population

Baseline characteristics	CVID patients (*n* = 39)	Controls (*n* = 30)	*P* value
Age mean ± SD (range)	49.8 ± 12 (21–69)	49.7 ± 13 (25–69)	0.87^a^
Male, *n* (%)	15 (38)	11 (37)	0.99^b^
BMI mean ± SD (range)	26.0 ± 4.7 (16.9–38.3)	24.4 ± 3.2 (19.5–31.0)	0.20^a^
Smoking, *n* (%)	4 (10)	2 (7)	0.99^b^
Statins, *n* (%)	3 (0.08)	0	0.25^b^
Infection only, *n* (%)	7 (18)	-	-
Enteropathy, *n* (%)	13 (33)	-	-
Lymphoid hyperplasia, *n* (%)	23 (59)	-	-
Granulomas, *n* (%)	6 (15)	-	-
Organ-specific autoimmunity, *n* (%)	8 (21)	-	-
Autoimmune cytopenia, *n* (%)	8 (21)	-	-
Bronchiectasis, *n* (%)	13 (33)	-	-

*BMI* body mass index. Data were analyzed using ^a^Mann-Whitney *U* test or ^b^Fisher’s exact test. Information on smoking habits was missing in three controls

**Table 2:** Plasma proportions of *n-3* PUFAs, *n-6* PUFAs, and MUFAs in CVID patients and healthy controls

	CVID patients (***n*** = 39)	Controls (***n*** = 30)
*n-3* PUFAs		
C18:3*n-3* (ALA)*	**0.83 (0.73–0.92)**	0.74 (0.54–0.84)
C18:4*n-3* (SDA)*	**0.04 (0.03–0.06)**	0.03 (0.02–0.04)
C20:4*n-3*	0.17 (0.14–0.21)	0.16 (0.13–0.19)
C20:5*n-3* (EPA)*	1.40 (0.95–1.79)	**1.93 (1.21–2.48)**
C21:5*n-3*	0.010 (0.005–0.018)	0.017 (0.005–0.028)
C22:5*n-3* (DPA)	0.75 (0.63–0.83)	0.78 (0.65–0.90)
C22:6*n-3* (DHA)*	3.15 (2.42–3.92)	**3.52 (2.93–4.33)**
*n-6* PUFAs		
C18:2*n-6* (LA)****	24.5 ± 3.30	**28.1 ± 2.70**
C18:3*n-6* (GLA)	0.39 (0.31–0.48)	0.33 (0.20–0.45)
C20:2*n-6*	0.21 ± 0.04	0.19 ± 0.03
C20:3*n-6* (DGLA)**	**1.68 ± 0.29**	1.49 ± 0.30
C20:4*n-6* (ARA)	7.15 ± 1.31	6.72 ± 1.41
C22:2n-6	0.01 (0.009–0.012)	0.01 (0.008–0.012)
C22:4n-6****	**0.21 ± 0.04**	0.16 ± 0.05
C22:5n-6**	**0.12 (0.10–0.15)**	0.10 (0.08–0.12)
MUFAs		
C14:1*n-5*	0.05 (0.03–0.08)	0.04 (0.04–0.07)
C16:1*n-7*	1.61 ± 0.54	1.57 ± 0.40
C16:1n-9***	**0.27 ± 0.07**	0.22 ± 0.03
C18:1n-7**	**1.66 ± 0.25**	1.49 ± 0.15
C18:1n-9****	**22.2 ± 3.29**	18.4 ± 2.45
C20:1*n-7*	0.013 (0.011–0.017)	0.014 (0.011–0.015)
C20:1n-9**	**0.19 (0.17–0.22)**	0.15 (0.13–0.18)
C20:1*n-11*	0.04 (0.03–0.05)	0.04 (0.03–0.05)
C22:1*n-7*	0.010 ± 0.003	0.010 ± 0.002
C22:1n-9***	**0.04 (0.03–0.04)**	0.03 (0.03–0.04)
C22:1*n-11*	0.005 (0.002–0.020)	0.007 (0.003–0.015)
C24:1*n-9*	1.34 ± 0.31	1.31 ± 0.24

*CVID* common variable immunodeficiency, *ALA* alpha-linolenic acid, *SDA* stearidonic acid, *EPA* eicosapentaenoic acid, *DPA* docosapentaenoic acid, *DHA* docosahexaenoic acid, *LA* linoleic acid, *GLA* gamma-linolenic acid, *DGLA* dihomo-gamma-linolenic acid, *ARA* arachidonic acid, *MUFAs* monounsaturated fatty acids, *PUFAs* polyunsaturated fatty acids. Values indicate plasma FA content by weight percent (wt%). FAs significantly changed are marked in bold, as well as the corresponding mean/median values that are increased in either CVID or controls

Data were analyzed using Student’s *t*-test or Mann–Whitney *U* test, as appropriate, and are presented as mean ± SD or median (25–75 percentile), respectively. *P < 0.05, **P < 0.01, ***P < 0.001, ****P < 0.0001

In contrast, the essential FA alpha-linolenic acid (ALA, C18:3*n-3*, *P* < 0.05), as well as the downstream FA C18:4*n-3* (stearidonic acid, SDA, P < 0.05) were increased in CVID as compared to controls (Table 2). These findings are not conflicting with the above results (reduced EPA and DHA) since studies have shown that the conversion of ALA to EPA, and in particular to DHA, is very inefficient in humans (Fig. 1) [27, 28].

### Reduced *n-6* PUFAs in CVID

Plasma proportion *of n-6* PUFAs was reduced in CVID compared to healthy controls (*P* < 0.001, Fig. 2B). This observation was primarily caused by a lower percentage of C18:2*n-6* (linoleic acid, LA, *P* < 0.0001, Table 2), which is the main contributor of *n-6* PUFA levels and constitutes an essential FA not synthesized by humans (Fig. 1). In contrast, other downstream *n-6* PUFA metabolism products were increased in CVID, with increased proportions of C20:3*n-6* (dihomo-gamma-linolenic acid, DGLA, *P* < 0.01), C22:4*n-6* (*P* < 0.0001), and C22:5*n-6* (*P* < 0.01, Table 2). Notably, recent studies support that individual *n-6* PUFAs, and in particular LA, may have anti-inflammatory properties, which could be beneficial to health [11, 29–33].

### Monosaturated FAs (MUFAs), but Not Saturated FAs (SFAs) and Trans FAs, Were Increased in Patients with CVID

MUFAs are either obtained from the diet or synthesized by elongase/desaturase enzymes from SFAs. Plasma MUFAs were increased in CVID as compared to healthy controls (*P* < 0.0001, Fig. 2C), mainly due to increased C18:1*n-9* (*P* < 0.0001), which is the major contributor to MUFA levels measured in humans, and increased C18:1*n-7* (*P* < 0.01, Table 2). However, the effects of altered MUFAs on health and disease are at present not clear [34].

Plasma FA proportions of total SFAs (*P* = 0.76), free FAs (*P* = 0.06), and trans FAs (*P* = 0.18) were not different between CVID patients and healthy controls (Supplemental Table S1).

### FA Composition Is Stable Over Time in CVID Patients

To study if plasma FA proportions of *n-3* PUFAs, *n-6* PUFAs and MUFAs remained stable over time, we measured these FAs in 20 CVID patients (11 females, mean age 50.8 years, SD 6.9) at three different time points over 8 weeks (0, 2, and 8 weeks). Sixteen patients completed all three measurements, and we found no significant changes in plasma wt% of *n-3* PUFAs (*P* = 0.27), *n-6* PUFAs (*P* = 0.67), or MUFAs (*P* = 0.53) during these measurements (Supplemental Fig. S1), suggesting that intra-individual FA profiles are relatively stable in CVID patients.

### CVID Patients Have a Potential Unfavorable FA Composition

To evaluate the potential net effects of the PUFA pattern in the CVID patients, we calculated the anti-inflammatory index based on the proportions of the potential anti-inflammatory FAs (DPA + DHA + DGLA + EPA), relative to the potential inflammatory FA ARA, showing a significant reduction in the anti-inflammatory index in CVID as compared to controls (*P* = 0.02, Fig. 2D). Furthermore, the *omega 3* index (the sum of EPA and DHA, expressed as a percentage of the total FA content) was also reduced in patients with CVID (Fig. 2E). Taken together, these results may suggest that CVID patients have an unfavorable FA composition with potential pro-inflammatory net effects.

### Associations Between Dietary and Plasma *n-3* and *n-6* PUFAs in CVID Patients

We next examined the possible contribution of diet and FA gut absorption to the disturbed FA profile in CVID. Some plasma FAs are commonly used as objective indicators of dietary FA intake, e.g., ALA and LA, which are not biosynthesized at all, and EPA and DHA, which are synthesized endogenously in very limited amounts (Fig. 1) [35, 36]. In the CVID patients (*n* = 37), dietary intake of the marine FAs, EPA, and DHA were positively correlated with their respective plasma values (EPA; rho = 0.41, *P* < 0.05, and DHA; rho = 0.47, *P* < 0.01, respectively). Contrary, dietary intake of the *n-3* PUFA ALA did not correlate with plasma ALA (rho = 0.21, *P* = 0.22). These data are consistent with prior publications showing significant correlations between dietary EPA and DHA, and their respective plasma FAs, whereas data on the relation between dietary and plasma ALA are less consistent [35–38]. Interestingly, we found no significant correlation between dietary and plasma LA (rho = 0.30, *P* = 0.07) in CVID patients, even though previous studies have reported positive associations between dietary and plasma levels of LA [35, 39, 40]. This may imply that absorption or other factors are affecting the conversion from dietary LA to plasma LA in CVID.

Notably, the mean fish intake in this CVID cohort was 81 g/day, and according to National Dietary Survey [41], mean Norwegian dietary fish intake was 67 g/day, suggesting that the reduced marine *n-3* PUFAs, EPA, and DHA, in CVID patients, were not merely related to reduced fish intake from the diet (Supplemental Fig. S2A). Overall, there were no differences in neither the dietary intake of fat, protein, carbohydrate, meat and egg, nor the dietary intake of SFAs, MUFAs, and total PUFAs, between CVID patients and the general Norwegian population, according to Norkost 3 (Supplemental Fig. S2B-C), indicating a normal diet in the CVID patients compared to the general Norwegian population.

### Gut Microbiome and FA Profile in CVID Patients

We have previously reported reduced microbial diversity and gut microbial dysbiosis in CVID patients compared to controls, which was not related to use of antibiotics [5]. When evaluating microbial diversity and its association to FAs in patients with CVID, multiple measures of intra-individual alpha diversity were positively associated with plasma *n-6* PUFAs (Faith’s PD whole tree index, *r* = 0.41, *P* < 0.001 and Supplemental Table S2), but not plasma *n-3* PUFAs (Faith’s PD whole tree, *r* = 0.05, P = 0.78 and Supplemental Table S2). Furthermore, LA, which constitutes the main contributor of *n-6* PUFA levels and has potential beneficial health benefits [30], also correlated positively with alpha diversity (Faith’s PD whole tree, *r* = 0.51, *P* < 0.001 and Supplemental Table S2). In contrast, plasma MUFAs were negatively associated with microbial diversity (Faith’s PD whole tree, *r* =  − 0.34, *P* = 0.036, Supplemental Table S2).

Next, we examined if 15 specific bacterial taxa, previously found to differentiate between CVID patients and healthy controls [5, 42], were associated with plasma proportions of MUFAs, *n-3*, and *n-6* PUFAs (Table 3). *Desulfovibrionaceae*, shown to be decreased in CVID patients, was negatively correlated with *n-3* PUFAs and EPA and showed a positive association with MUFAs. No other significant correlations between the FA pattern and the 15 selected bacterial taxa were found (Table 3).

**Table 3:** Correlations between *n-3* PUFAs, *n-6* PUFAs and MUFAs, and bacterial taxa involved in gut microbial dysbiosis in CVID

Bacterial taxa	Increased in:	Correlation with *n-3* PUFAs, rho	Correlation with EPA, rho	Correlation with DHA, rho	Correlation with *n-6* PUFAs, rho	Correlation with MUFAs, rho
*Bacilli*^a^	CVID	− 0.01	0.01	− 0.04	0.07	− 0.12
*Dorea*^a^	CVID	− 0.10	− 0.12	− 0.12	0.22	0.01
*Roseburia*^a^	CVID	0.11	0.08	0.11	− 0.31	0.10
*Gammaproteobacteria*^a^	CVID	−0.01	− 0.10	0.03	− 0.10	0.01
*Hungatella*^*b*^	CVID	0.21	0.13	0.26	0.013	− 0.08
*Flavonifractor*^b^	CVID	− 0.01	− 0.03	− 0.02	− 0.15	0.23
*Veillonella*^b^	CVID	0.03	− 0.02	0.03	− 0.23	0.15
*Escherichia-Shigella*^b^	CVID	− 0.11	− 0.18	− 0.10	− 0.04	− 0.04
*Bifidobacterium*^a^	Healthy	0.12	0.27	− 0.04	0.08	0.03
*Porphyromonadaceae*^*a,c*^	Healthy	− 0.07	− 0.11	0.07	0.13	− 0.04
*Christensenellaceae*^a^	Healthy	− 0.17	− 0.13	− 0.16	0.30	− 0.25
*Blautia*^a^	Healthy	0.11	0.07	0.15	0.00	0.08
*Sutterella*^a^	Healthy	0.17	0.16	0.23	− 0.11	0.17
*Desulfovibrionacea*^a^	Healthy	− 0.34*	− 0.32*	− 0.25	− 0.18	0.37*
*Christensenellaceae R-7 group*^*b*^	Healthy	− 0.12	− 0.16	− 0.18	0.29	− 0.26

*CVID* common variable immunodeficiency, *EPA* eicosapentaenoic acid, *DHA* docosahexaenoic acid, *MUFAs* monounsaturated fatty acids, *PUFAs* polyunsaturated fatty acids. For the individual plasma FAs, we only studied those that are synthesized endogenously in limited amounts/not synthesized at all and were found to correlate with diet, i.e., EPA and DHA. Correlations were calculated by the Spearman’s rank correlation test and are presented by rho. Significant correlations are marked in bold. *P < 0.05, **P < 0.01, ***P < 0.001

^a^Significant bacterial taxa from Jørgensen et al., *Mucosal Immunol* 2016 [5]

^b^Significant taxa from Jørgensen et al., *J Allergy Clin Immunol*, 2019 [42]

^c^Formally known as Odoribacteraceae

### Rifaximin-Induced Alterations on Plasma FAs

Rifaximin is a broad-spectrum oral antibiotic with negligible systemic absorption, making it suitable to study how changes in gut microbial composition affect systemic markers in blood. We have previously shown that a 2-week course of rifaximin led to significant alterations in gut microbial composition in CVID with a decrease in alpha diversity and alteration in 16 different bacterial taxa [15]. In the present study, we explored if rifaximin-induced alterations of gut microbial composition could influence plasma FA proportions. Briefly, 39 CVID patients, aged 21–69 years (62% women), were randomized to rifaximin 550 mg bid versus no treatment for 2 weeks, and followed up for an additional 6 weeks. When comparing the two groups (*rifaximin* versus *no treatment*), we found a significant change of the proportion of *n-6* PUFAs (*P* = 0.04, UNIANOVA), due to a significant decrease from week 0 to week 2 in the patients receiving rifaximin (Table 4). This change was also significant for the major contributor of *n-6* PUFAs, LA (Table 4). However, there was no significant change in the plasma FA proportions of neither *n-3* PUFAs nor EPA and DHA (Table 4). The proportion of plasma MUFAs increased significantly from week 0 to week 2 in the *rifaximin* arm compared to the *no treatment* arm, but the impact of rifaximin did not last to the end of the observation period (Table 4).

**Table 4:** The effect of rifaximin on fatty acids

Fatty acids	Group	Baseline^a^	2 weeks^a^	8 weeks^a^	*P* value^b^ interaction
***n-3* PUFAs** (wt%)	No Int	6.4 (5.5–7.8)	6.0 (5.0–7.7)	6.2 (5.6–7.3)	0.587
	Rif	5.8 (5.1–7.1)	6.4 (4.8–7.2)	6.5 (5.3–7.7)	
EPA (wt%)	No Int	1.6 (1.1–1.9)	1.4 (0.7–1.9)	1.4 (1.2–1.8)	0.403
	Rif	1.2 (0.9–1.7)	1.2 (0.8–1.9)	1.5 (1.0–2.0)	
DHA (wt%)	No Int	3.2 (2.6–4.0)	3.3 (2.4–3.9)	3.0 (2.6–3.8)	0.491
	Rif	2.8 (2.0–3.6)	2.8 (2.1–3.7)	3.2 (2.4–3.7)	
***n-6* PUFAs** (wt%)	No Int	33.3 (31.0–36.7)	34.8 (31.0–37.3)	35.0 (30.9–37.5)	0.040
	Rif	35.3 (32.7–37.0)	33.2 (30.0–36.0)**	32.7 (29.6–35.7)	
LA (wt%)	No Int	22.9 (20.8–26.8)	24.4 (22.2–28.1)	25.8 (21.8–27.8)	0.035
	Rif	24.8 (23.4–27.1)	23.5 (20.9–26.0)**	23.6 (21.5–27.1)	
**MUFAs** (wt%)	No Int	27.8 (25.7–28.7)	26.8 (24.6–27.5)	28.3 (26.1–30.4)	0.087
	Rif	26.8 (25.8–30.3)	27.6 (25.8–30.7)*	28.2 (25.4–31.2)	

*EPA* eicosapentaenoic acid, *DHA* docosahexaenoic acid, *MUFAs* monounsaturated fatty acids, *PUFAs* polyunsaturated fatty acids, *LA* linoleic acid. Fatty acid proportions in plasma in the “rifaximin” (Rif, *n* = 19) and in the “no intervention” group (No Int, *n* = 20)

^a^Data are given in median (25–75 percentile)

^b^The *P* value reflects the interaction between time and group from UNIANOVA

^*^P < 0.05 vs. baseline, **P ≤ 0.01 vs. baseline

Based on these findings, we wanted to explore if there were any correlations between the 16 bacterial taxa that we have previously shown to be significantly changed by a 2-week course with rifaximin [15] and FAs altered by rifaximin (Supplemental Table S3). We found that the decrease in plasma FA proportion of *n-6* PUFAs, after rifaximin therapy, was positively correlated with two taxa from the Firmicutes phyla: *Ruminococcaceae UCG-002* (rho = 0.44, *P* < 0.01) and *Clostridiales*. *Family XIII.Family XIII UCG-001* (rho = 0.41, *P* < 0.01). These bacterial taxa also correlated with the major contributor to *n-6* PUFAs, LA (Supplemental Table S3). Interestingly, the increase in MUFAs from week 0 to week 2 could be related to a negative correlation with the same taxon that was positively correlated with *n-6* PUFAs, namely *Ruminococcaceae UCG-002* (rho =  − 0.488, *P* < 0.01).

To summarize the gut microbiota-related findings, *n-6* PUFAs and LA correlated with alpha diversity (reflecting microbial diversity), and all three measures were reduced by rifaximin therapy. Two bacteria that were decreased by rifaximin also correlated with *n-6* PUFAs, potentially suggesting a link between alpha diversity, *n-6* PUFAs and specific taxa.

### FAs and Clinical Subgroups in CVID

We evaluated whether the FA profile in CVID could be related to different clinical subgroups of CVID patients. There were no significant differences in plasma FA proportions of *n-3* PUFAs, *n-6* PUFAs, and MUFAs between CVID patients with non-infectious complications (*n* = 32) compared to CVID patients with infection only (*n* = 7) (Supplemental Table S4). Furthermore, when categorizing CVID patients according to presence or absence of specific clinical complications, such as enteropathy, splenomegaly, lymphoid hyperplasia, granulomas, organ-specific autoimmunity, autoimmune cytopenia, or bronchiectasis, we found no differences in the proportions of plasma *n-3* PUFAs, *n-6* PUFAs, and MUFAs between groups (Supplemental Table S4).

### FAs Are Associated with Serum IgG Levels

All CVID patients were on Ig substitution (six on intravenous Ig (IVIG), 30 on subcutaneous Ig (SCIG), and three on both IVIG and SCIG). The median serum IgG level in the CVID population was 7.7 g/L (IQR 2.6 g/L, range 1.5–15.1 g/L). Interestingly, IgG levels correlated with plasma *n-3* PUFAs (rho = 0.36, *P* = 0.03), DHA (rho = 0.41, *P* = 0.009), and *omega 3* index (rho = 0.38, *P* = 0.02), but not *n-6* PUFAs (rho = 0.07, *P* = 0.66) or MUFAs (rho = -0.21, *P* = 0.20).

In contrast, except for higher proportions of *n-3* PUFAs in relation to increased proportion of transitory B cells, we found no associations between the FA profiles and any of the other examined B cell subpopulations (Supplemental Table S5), or with the absolute levels of CD3^+^ T cells, CD4^+^ T cells, or CD8^+^ T cells (Supplemental Table S6).

## Discussion

To the best of our knowledge, this is the first study investigating the FA profile in CVID patients. Our main findings in this pilot study were as follows: (i) CVID patients have a potential unfavorable FA profile with reduced plasma FA proportions of EPA and DHA and a reduced anti-inflammatory index; (ii) *n-6* PUFAs and LA were decreased in CVID patients and were positively associated with gut microbial alpha diversity; (iii) a 2-week course of oral rifaximin known to reduce gut microbial diversity led to a reduction of *n-6* PUFAs; (iv) serum IgG levels were positively associated with plasma *n-3* PUFAs, DHA, and *omega 3* index; (v) the altered plasma FA proportions of *n-3* and *n-6* PUFAs did not seem to be related to altered dietary intake, intestinal absorption, or the presence of CVID enteropathy.

There are numerous reports on the potential beneficial effects of increased *n-3* PUFAs on autoimmunity and metabolic disorders such as diabetes, obesity, and cardiovascular diseases [20, 43, 44], supporting that reduced plasma proportions of EPA and DHA and a reduced anti-inflammatory FA index in CVID patients may represent an unfavorable FA profile. In contrast, health benefits of total and individual *n-6* PUFAs are conflicting and appear less clear [45–48]. Indeed, recent epidemiological studies have challenged the concept of inflammatory and harmful effects of *n-6* PUFAs, including LA [11, 29–33]. However, further studies are needed to elucidate the effect of decreased levels of *n-6* PUFAs and LA, particularly in CVID.

CVID enteropathy did not influence plasma FA percentage in the CVID patients, suggesting that altered FA profile between controls and patients with CVID was not due to enteropathy-associated malabsorption. Furthermore, in line with previous studies, we found positive correlations between dietary sources of the marine *n-3* PUFAs, EPA, and DHA, and their respective plasma proportions [35, 36, 38] as well as increased rather than decreased intake of these FAs in CVID as compared to controls, suggesting that the altered levels of *n-3* PUFAs, EPA, and DHA do not merely reflect altered intake or malabsorption. The lack of correlation between dietary and plasma proportion of ALA is, however, in line with considerable discrepancies in previous published studies [35–38]. The fact that dietary LA and plasma LA did not correlate in CVID may be related to gut microbial modulation as discussed below.

Several studies, both in mice and humans, suggest that the gut microbiota could play a role in lipid metabolism and lipid levels in blood and tissues [49]. Although several intervention studies have been conducted to assess the impact of dietary PUFAs, SFAs, and MUFAs on gut microbiota composition [50–52], studies on the relationship between gut microbiota and blood PUFAs and MUFAs are rather scarce both in healthy subjects and in disease states [53]. In the present study, *Desulfovibrionaceae* in stools correlated negatively with plasma proportions of *n-3* PUFAs and EPA and positively with MUFAs. This bacterial family has previously been shown to be reduced in CVID and is a part of the CVID dysbiosis index, and although the implication of this finding is at present unclear, the negative association between *n-3* PUFAs and positive association with MUFAs merit further investigation.

A major finding in the present study was a significant association between *n-6* PUFAs and LA and the gut microbial intra-individual alpha diversity in CVID patients. This association was further supported when microbial therapy with rifaximin both decreased *n-6* PUFAs and LA and, as previously shown, gut microbial alpha diversity. Interestingly, specific taxa, which were altered by rifaximin, correlated with *n-6* PUFAs, linking specific gut microbial bacterial taxa to plasma *n-6* PUFAs. Hence, the findings support an important “proof of concept” that alteration of the gut microbial composition could affect FA measurements in blood. Whereas most studies on FAs and gut microbiota have been focusing on *n-3* PUFAs, our findings suggest that *n-6* PUFAs, and particular LA, could be of relevance in relation to the interaction between gut microbiota and FAs.

The fact that serum IgG levels correlated with a favorable FA profile (i.e., *n-3* PUFAs, DHA, and the *omega 3* index) suggests an interaction between these FAs and an important, and correctable, immunological feature of CVID. A recent animal study found that appropriate absorption of dietary EPA and DHA from the gut were dependent on presence of plasma IgG [54]. These observations should be further evaluated in humans but offers an interesting hypothesis regarding the link between favorable FA, gut absorption, and immunological features in CVID.

The strengths of this study are, compared to other FA studies, the inclusion of detailed dietary and gut microbial data. In addition, we report results from a randomized control trial evaluating the effect of microbial therapy on plasma proportions of FAs in CVID patients. Finally, the longitudinal data on MUFAs, *n-3*, and *n-6* PUFAs in patients with CVID confirmed stable FA proportions over time, implying that FAs are relative stable markers in this cohort. The study also has several limitations. Relatively few patients and controls were examined, particularly in relation to clinical sub-group analyses. The fact that only seven patients were classified in the less inflammatory sub-group (“infection only”) may have contributed to the lack of statistical significance in the FA profiles when compared to the more inflammatory subgroup (“complications”). Moreover, we cannot exclude that a longer intervention with rifaximin could have resulted in more changes to the gut microbial composition, as well as in alterations of the plasma FAs, and the data from this pilot study should be confirmed in a larger study population with a longer duration of intervention. Furthermore, we did not have diet data on the healthy controls and therefore used data from National Dietary Survey as a control cohort for dietary analyses. Also, many comparisons were performed, and some of the findings could be by chance. Finally, correlations do not necessarily prove any causal relationship, and the data from this study should be further examined in more mechanistic studies.

In conclusion, we found a potentially unfavorable FA profile in CVID patients with reduced plasma EPA, DHA, and anti-inflammatory index and decreased *n-6* PUFAs and LA. Plasma FA proportions of *n-6* PUFAs and LA were related to gut microbial diversity and altered by a 2-week course of rifaximin, suggesting an intriguing interaction between gut microbiota and these FAs, and in particular LA, in CVID. These findings should be confirmed and further investigated in a larger population, particularly in relation to subgroup analyses. Future studies should be conducted to investigate the relationship between gut microbiota and the FA profile at a mechanistic level, as well as the potential clinical consequences of an unfavorable FA composition in CVID.

## Supplementary Information

Below is the link to the electronic supplementary material.

Supplementary file1 (DOCX 345 KB)

## Data Availability

The datasets analyzed during the current study are not publicly available due to Norwegian legislation regarding general data protection regulation but are available from the corresponding author (TS), on reasonable request.

## Abbreviations

ALA: Alpha-linolenic acid
ARA: Arachidonic acid
CVID: Common variable immunodeficiency
D5D: Delta-5 desaturase
D6D: Delta-6 desaturase
DGLA: Dihomo-gamma-linolenic acid
DHA: Docosahexaenoic acid
DPA: Docosapentaenoic acid
EPA: Eicosapentaenoic acid
FA: Fatty acid
GLA: Gamma-linolenic acid
Ig: Immunoglobulin
LA: Linoleic acid
MUFAs: Monounsaturated fatty acids
PUFAs: Polyunsaturated fatty acids
SDA: Stearidonic acid
