# Supplementary material for: Altered Plasma Fatty Acids Associate with Gut Microbial Composition in Common Variable Immunodeficiency
Source: J Clin Immunol. 2021 Oct 20;42(1):146–57. doi: 10.1007/s10875-021-01146-9 (PMC8821409; doi:10.1007/s10875-021-01146-9)
Supplement: Supplementary file 1 — Supplementary file1 (DOCX 345 KB) [file 10875_2021_1146_MOESM1_ESM.docx]

**Supplemental information**

**Altered plasma fatty acids associate with gut microbial composition in Common variable immunodeficiency**

Tonje Skarpengland, Magnhild E. Macpherson, Johannes R. Hov, Xiang Y. Kong, Pavol Bohov, Bente Halvorsen, Børre Fevang, Rolf K. Berge, Pål Aukrust, Silje F. Jørgensen

**Supplemental Methods**

**Supplemental Tables**

**Supplemental Figures**

**Supplemental methods**

*Gut microbiota analyses*

The sequencing was performed on an Illumina MiSeq. Briefly, the hypervariable V3-V4 region was amplified with generic primers as described in detail [1], including the gene specific primer sequences CCTACGGGAGGCAGCAG (forward) and GGACTACHVGGGTWTCTAAT (reverse) and up to 192 (24x8 barcodes) samples in parallel. Cleanup and normalization were performed using the SequalPrep Normalization Plate Kit (Life Technologies), followed by pooling and quality control. The final libraries were sequenced on an Illumina MiSeq (Norwegian Sequencing Centre, Oslo University Hospital Ullevål) using the v3 kit, allowing up to 300 basepairs paired-end sequencing. Paired-end reads were quality trimmed with cutadapt version 1.13 and then merged using FLASH version 1.2.11. The merged reads were de-multiplexed and quality filtered using default values in Quantitative Insights Into Microbial Ecology (QIIME) version 1.9.1. Closed reference operational taxonomic unit (OTU) mapping to the Silva database (version 123, reference OTUs clustered at 97% sequence similarity) was performed using SortMeRNA version 2.0 through QIIME. A rarefied OTU table (9525 reads per sample) was generated and OTUs with less than 2 reads to support it were discarded.

*Rifaxmin study*

The baseline cohort is the same cohort/samples as previously described in this paper (n=39). These CVID patients were randomized in an open, prospective, single-center, clinical trial (the rifaximin study) at Oslo University Hospital, Rikshospitalet, Norway, between Oct 8 2013, and Oct 20, 2014 [2]. In the present study, there was one individual less (n=39) compared to the original rifaximin publication (n=40) because there was not enough plasma available for FA analyses. The CVID patients were randomized by computer-generated randomization (1:1) into two groups (*rifaximin n=19* and *no intervention n=20*) and there was no placebo drug [2].

**Supplemental Figure S1**

**A**


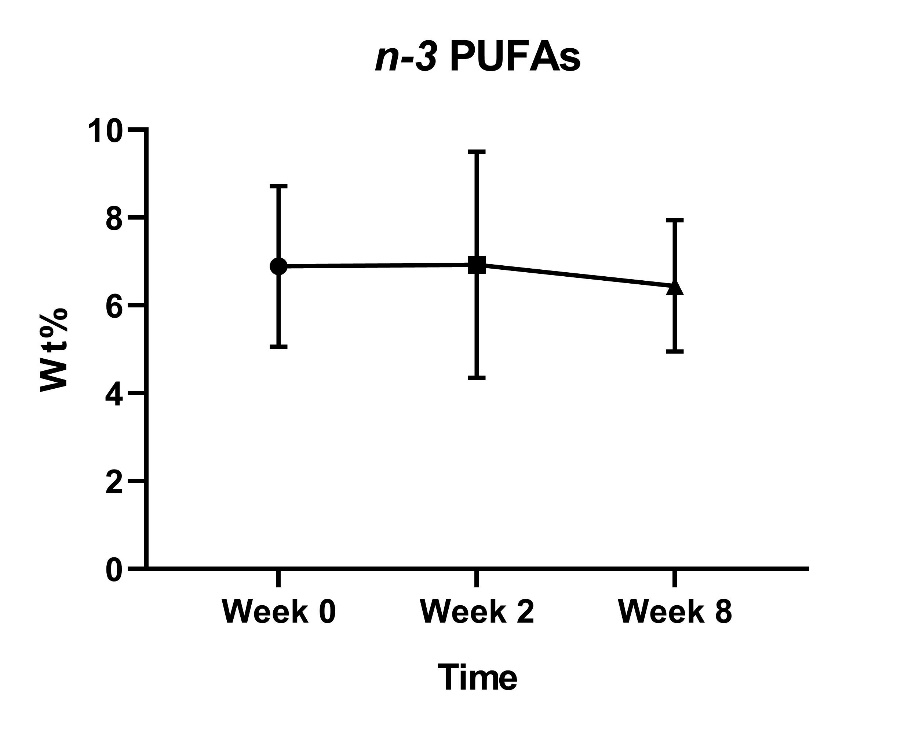


**B**


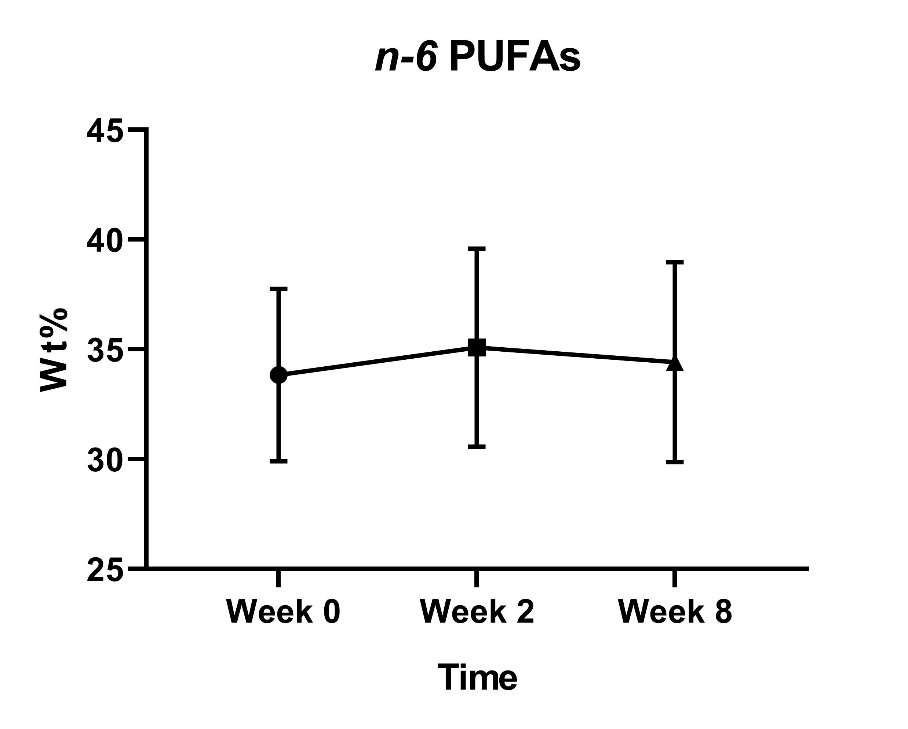


**C**


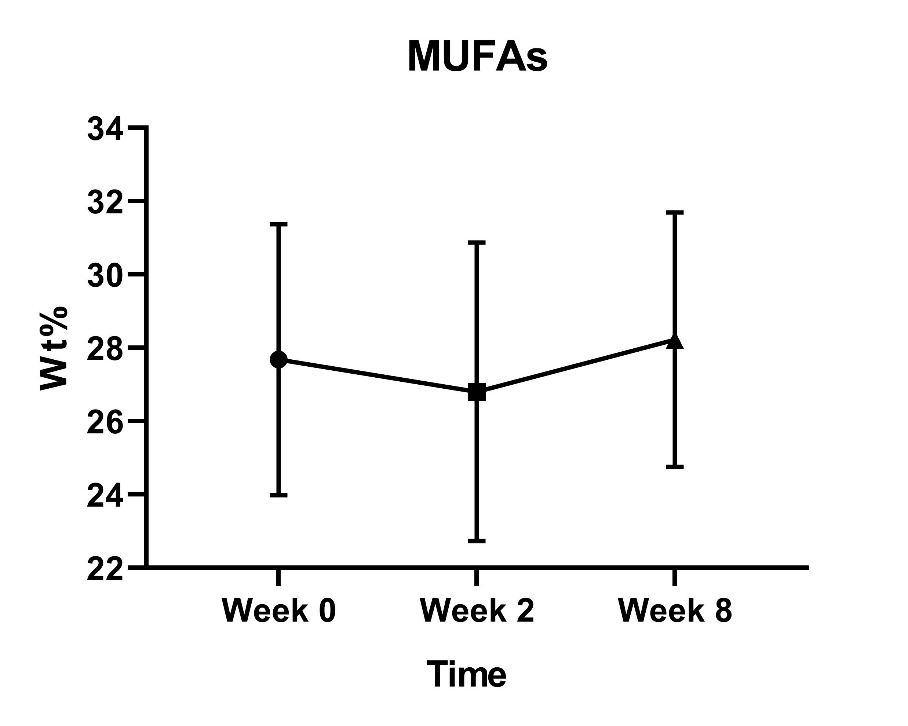


**Supplemental Figure S1:** Temporal testing of *n-3* PUFAs, *n-6* PUFAs and MUFAs. Plasma FA proportions over three time points (0, 2 and 8 weeks) for (A) *n-3* PUFAs, (B) *n-6* PUFAs, (C) MUFAs in CVID patients (n=16). P-values were calculated using the ANOVA test or Friedman’s test as appropriate. No significant differences were detected. Results are shown as mean with SD. PUFAs, polyunsaturated fatty acids; MUFAs, monounsaturated fatty acids; Wt%, weight %.

**Supplemental Figure S2**

**A**


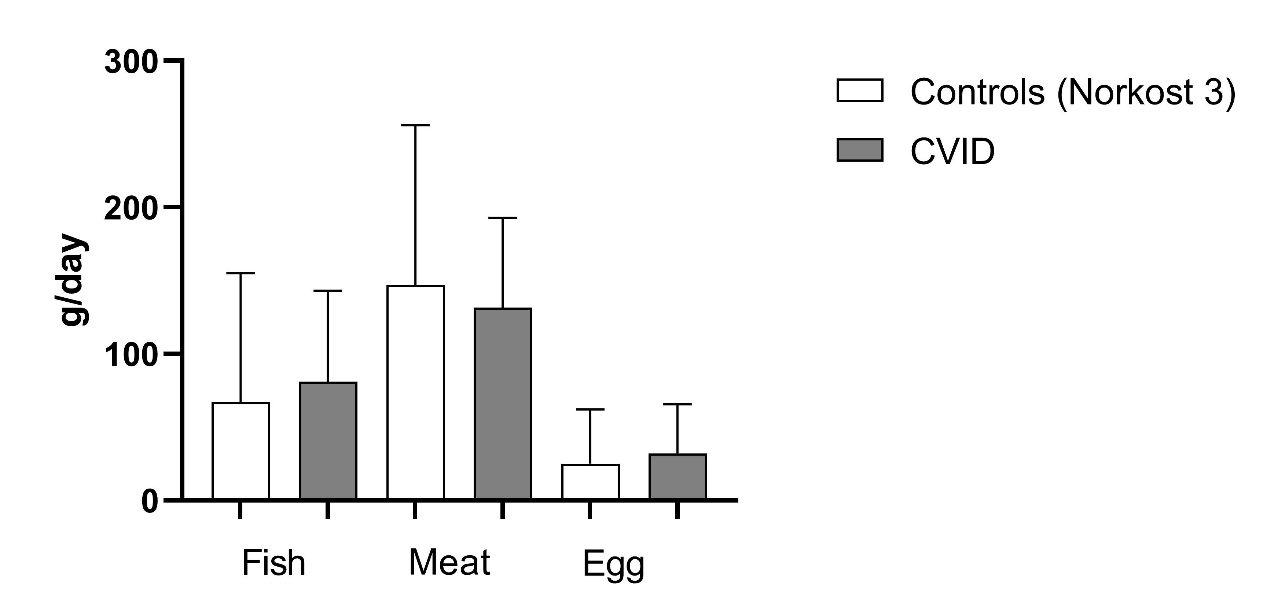


**B**


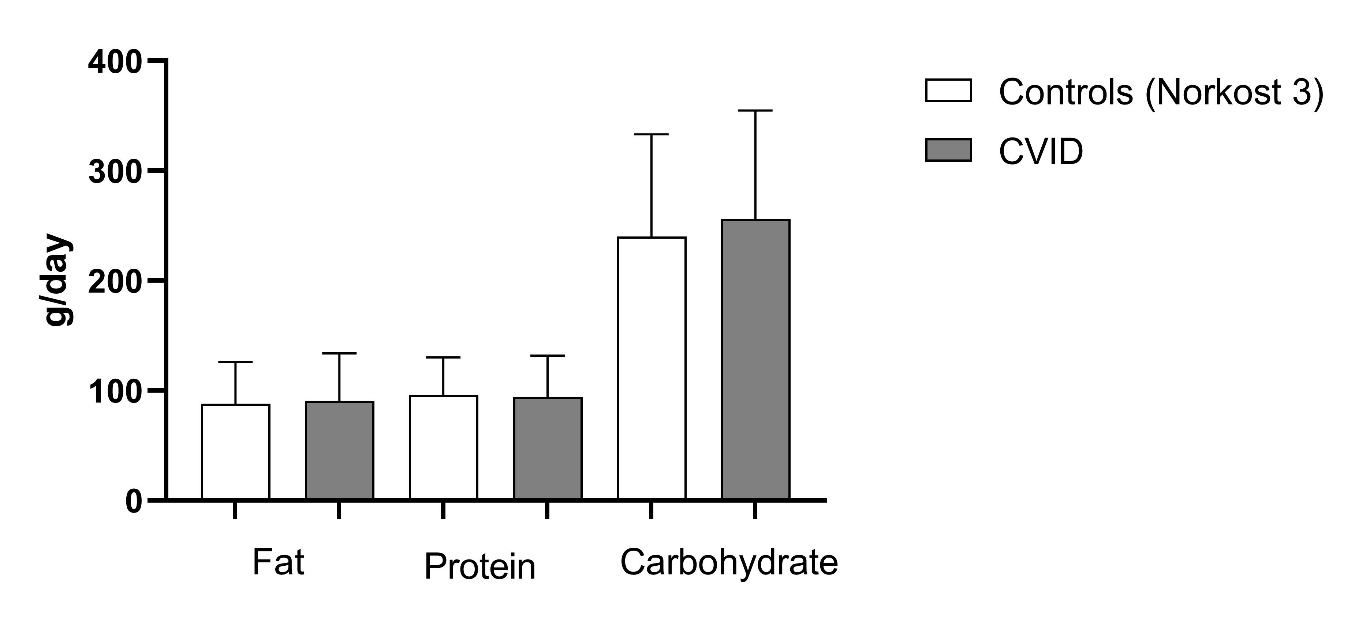


**C**

**
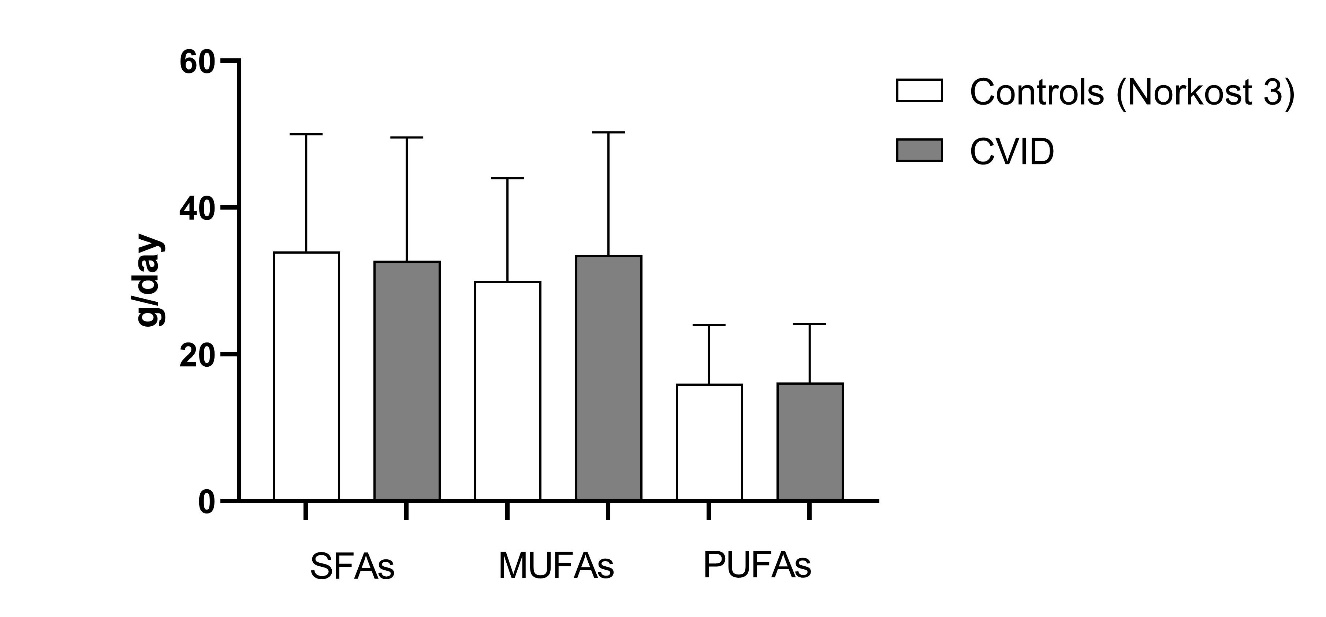
**

**Supplemental Figure S2:** Daily intake of fish and macronutrients in the CVID patient cohort (n=37) versus the Norwegian background population (Norkost3, n=1787). Results are shown as mean and SD. We found no differences between the groups when applying t-test analyses for intake of fish (P=0.34), meat (P=0.39), egg (P=0.25), protein (P=0.72), carbohydrate (P=0.30) and fat (P=0.68). Daily intake of fat (P=0.68), protein (P=0.72), carbohydrate (P=0.30), SFAs (P=0.34), MUFAs (P=0.13) and PUFAs (P=0.88) were also similar. Dietary intake of *n-3* and *n-6* PUFAs were not available in Norkost 3 study. The Norkost 3 study used a 24-hour recall questionnaire, aiding accuracy of reported diet, whilst using a high ‘n’ to reduce the seasonal and day-to-day variation. PUFAs, polyunsaturated fatty acids; MUFAs, monounsaturated fatty acids; SFAs, saturated fatty acids.

**SUPPLEMENTAL TABLES**

**Supplemental Table S1. SFAs, free FAs and trans FAs in CVID patients and healthy controls**

|  | **CVID patients (N=39)** | **Controls (N=30)** |
| --- | --- | --- |
| SFAs | 31.1 ± 1.90 | 31.1 ± 1.60 |
| Free FAs | 0.28 (0.15-0.39) | 0.38 (0.22-0.57) |
| Trans FAs | 0.27 (0.22-0.33) | 0.29 (0.27-0.33) |

CVID, common variable immunodeficiency; SFAs, saturated fatty acids. Data were analysed using Student’s t-test or Mann-Whitney *U* test, as appropriate, and are presented as mean ± SD, or median (25–75 percentile), respectively. Values indicate plasma content by weight percent (wt %) [SFAs and trans FAs], and mmol/l [free FAs].

**Supplemental Table S2. Correlation between FAs and different alpha diversity measurements**

| **Alpha diversity measurements** | **Correlation with *n-3* PUFAs** | **Correlation with *n-6* PUFAs** | **Correlation with LA** | **Correlation with MUFA** |
| --- | --- | --- | --- | --- |
| Faith’s PD^a^ | 0.05 | **0.41***** | **0.51***** | **-0.34*** |
| Shannon^b^ | -0.23 | **0.45**** | **0.51***** | -0.29 |
| Chao1^a^ | 0.01 | **0.43**** | **0.50**** | **-0.34*** |

LA, linoleic acid; PUFAs, polyunsaturated fatty acids; MUFAs, monounsaturated fatty acids. ^a^Correlations were calculated by the Pearsons’ correlation test and are presented by Pearson’s *r*. ^b^Correlations were calculated by the Spearmans’ correlation test and are presented by rho. Significant correlations are marked in bold. * P<0.05, **P<0.01, ***P<0.001.

**Supplemental Table S3. Correlations between FAs and the 16 bacterial taxa that were significantly changed by rifaximin**

| **Taxa** | ***n-6* PUFAs** | **LA** | **MUFAs** |
| --- | --- | --- | --- |
| Bacteroidetes | 0.07 | 0.17 | -0.06 |
| Bacteroidetes.Bacteroidia | 0.07 | 0.17 | -0.06 |
| Bacteroidetes.Bacteroidia.Bacteroidales | 0.07 | 0.17 | -0.06 |
| Bacteroidetes.Bacteroidia.Bacteroidales.Bacteroidaceae | -0.16 | -0.12 | 0.16 |
| Bacteroidetes.Bacteroidia.Bacteroidales.Bacteroidaceae. Bacteroides | -0.16 | -0.12 | 0.16 |
| Firmicutes.Clostridia.Clostridiales.Family XI.Ezakiella | -0.20 | -0.04 | 0.17 |
| Firmicutes.Clostridia.Clostridiales.Family XIII.Family XIII UCG-001 | **0.41**** | **0.32*** | -0.28 |
| Firmicutes.Clostridia.Clostridiales.Lachnospiraceae. Anaerostipes | 0.12 | 0.20 | 0.11 |
| Firmicutes.Clostridia.Clostridiales.Lachnospiraceae. Lactonifactor | 0.05 | 0.12 | -0.01 |
| Firmicutes.Clostridia.Clostridiales.Peptococcaceae | 0.29 | **0.34*** | -0.28 |
| Firmicutes.Clostridia.Clostridiales.Ruminococcaceae | 0.32 | 0.19 | -0.29 |
| Firmicutes.Clostridia.Clostridiales.Ruminococcaceae. Anaerotruncus | 0.32 | 0.23 | -0.20 |
| Firmicutes.Clostridia.Clostridiales.Ruminococcaceae. Oscillibacter | 0.26 | 0.25 | **-0.37*** |
| Firmicutes.Clostridia.Clostridiales.Ruminococcaceae. Ruminococcaceae UCG-002 | **0.44**** | **0.42**** | **-0.49**** |
| Firmicutes.Clostridia.Clostridiales.Ruminococcaceae. Ruminococcaceae UCG-014 | 0.22 | 0.24 | -0.10 |
| Firmicutes.Clostridia.Clostridiales.Ruminococcaceae. uncultured | 0.24 | 0.13 | -0.22 |

Correlations were calculated by the Spearman's rank correlation test and are presented by rho. Significant correlations are marked in bold. *P<0.05, **P<0.01

**Supplemental Table S4. Fatty acid profile in different CVID phenotypes**

| **Phenotype** | ***n-3* PUFAs** | ***n-6* PUFAs** | **MUFAs** |
| --- | --- | --- | --- |
| Infections only | 0.57 | 0.19 | 0.76 |
| Enteropathy | 0.81 | 0.75 | 0.83 |
| Splenomegaly | 0.54 | 0.35 | 0.22 |
| Lymphoid hyperplasia | 0.71 | 0.27 | 0.66 |
| Granulomas | 0.33 | 0.72 | 0.97 |
| Organ-specific autoimmunity | 0.80 | 0.69 | 0.98 |
| Autoimmune cytopenia | 0.75 | 0.46 | 0.92 |
| Bronchiectasis | 0.57 | 0.09 | 0.17 |

PUFAs, polyunsaturated fatty acids; MUFAs monounsaturated fatty acids. Data were analysed using Student’s t-test or Mann-Whitney *U* test, as appropriate. Values indicate P value between CVID patients categorized according to presence or absence of the specified phenotype, respectively.

**Supplemental Table S5. Comparison of fatty acid in CVID, patients stratified by B cell subclasses**

| **Fatty acids** |  | | **Transitional B cell**: | | **CD21^low^ B cells** | | **Switched memory B cells:** | |
| --- | --- | --- | --- | --- | --- | --- | --- | --- |
|  |  |  | <9 %  (n=30) | ≥9%  (n=9) | <10 %  (n=18) | ≥10%  (n= 21) | ≤ 2%  (n=21) | >2%  (n=18) |
| ***n-3* PUFAs**  **mean±SD** |  |  | 6.13±1.23 | **7.75±2.48*** | ^a^5.93(1.41) | 6.43(2.89) | ^a^6.36(2.88) | 6.27(1.83) |
| **EPA**  **mean±SD** |  |  | 1.34±0.48 | **2.16±1.21**** | ^a^1.22(0.74) | 1.41(1.16) | ^a^1.40(0.95) | 1.47(0.88) |
| **DHA**  **mean±SD** |  |  | 2.95±0.87 | **3.76±1.33*** | 2.79±1.06 | 3.44±0.93 | 3.34±0.98 | 2.91±1.07 |
| ***n-6* PUFAs**  **mean±SD** |  |  | 34.4±3.63 | 34.0±2.33 | 34.2±2.97 | 34.4±3.72 | 33.9±3.43 | 34.7±3.30 |
| **MUFAs**  **mean±SD** |  |  | 28.0±3.55 | 26.5±2.85 | 27.8±2.74 | 27.5±3.98 | 27.9±3.26 | 27.4±3.68 |

CVID, common variable immunodeficiency; PUFAs, polyunsaturated fatty acids; MUFAs, monounsaturated fatty acids; EPA, eicosapentaenoic acid; DHA, docosahexaenoic acid. B cells are classified according to EUROclass. Analyses on the sub-classification of % CD19 B cells were not performed because there were only three individuals in % CD19 B cells < 1%. Data were analysed using Student’s t-test or Mann-Whitney *U* test, as appropriate. Values represent mean ± SD or ^a^median (IQR), as appropriate. Significant correlations are marked in bold. * P<0.05; **P<0.01.

**Supplemental Table S6. Correlation between fatty acids in CVID and absolute values of CD3, CD4 and CD8 T cells**

|  | **CD3 T cells**  **Rho** | **CD4 T cell**  **Pearson’s r** | **CD8 T cells**  **Rho** |
| --- | --- | --- | --- |
| MUFAs | -0.07 | -0.12 | -0.07 |
| *n-3* PUFAs | -0.13 | -0.47 | -0.2 |
| EPA | -0.14 | 0.03^a^ | -0.23 |
| DHA | -0.15 | -0.03 | -0.19 |
| *n-6* PUFAs | 0.13 | 0.04 | 0.19 |

MUFAs, monounsaturated fatty acids; PUFAs, polyunsaturated fatty acids; EPA, eicosapentaenoic acid; DHA, docosahexaenoic acid. Correlations were calculated by the Spearman's rank correlation test or Pearson correlation test, as appropriate, and are presented by rho or Pearson’s r, respectively. N=39 patients. ^a^Calculated by Spearman's rank correlation test and presented by rho.

**References**

1. Kozich, J.J., et al., *Development of a dual-index sequencing strategy and curation pipeline for analyzing amplicon sequence data on the MiSeq Illumina sequencing platform.* Appl Environ Microbiol, 2013. **79**(17): p. 5112-20.

2. Jørgensen, S.F., et al., *Rifaximin alters gut microbiota profile, but does not affect systemic inflammation - a randomized controlled trial in common variable immunodeficiency.* Sci Rep, 2019. **9**(1): p. 167.
